# Transient Hyperechogenic Medullary Pyramids in a Neonate With Acute Kidney Injury

**DOI:** 10.7759/cureus.47508

**Published:** 2023-10-23

**Authors:** Shubhi Gaur, Pratap H Parihar, Sheetal S Shelar, Harshith Gowda

**Affiliations:** 1 Department of Radiodiagnosis, Jawaharlal Nehru Medical College, Datta Meghe Institute of Higher Education and Research, Wardha, IND

**Keywords:** medulla, dehydration, ultrasound, acute kidney injury, neonate

## Abstract

A neonate with acute kidney injury can present with decreased urine output and signs of dehydration. Sonography is used to evaluate the kidneys for structural deformities. A normal sonographic image of a neonatal kidney would show hypoechoic pyramids of the medulla. However, less frequently occurring neonatal transient renal failure with renal medullary hyperechogenicity has been linked to severe perinatal renal damage, kidney abnormalities, or nephrocalcinosis.

A simple conventional sonography in neonates can be helpful in predicting the severity of renal damage in such cases. Hyperechogenecity of the medulla in contrast to the normal hypoechogenic medulla of normal neonates can be due to multiple causes. However one must bear in mind that this finding of hyperechoic tips of renal pyramids is not indicative of intrinsic renal disease and subsides without intervention if physiologic or with rehydration if due to hypernatraemic dehydration. It is important for a physician to know about this physiological variant seen in neonates who present with dehydration.

## Introduction

In the setting of a newborn intensive care unit (NICU), acute kidney injury (AKI) is frequently encountered and is linked to higher morbidity and fatality rates. Additionally, those who experience newborn AKI may be more likely to develop chronic kidney disease (CKD) [1]. Newborn AKI is categorized according to the Kidney Disease: Improving Global Outcomes (KDIGO) criteria. This definition should be used in research and clinical settings to diagnose and stage newborn AKI [2].

In a review article published by Starr et al. [3], the common risk factors for developing AKI included premature or low birth weight neonate, placental insufficiency, exposure to nephrotoxic medications (angiotensin-converting enzyme inhibitors, non-steroidal anti-inflammatory drugs), delivery complications resulting in hypoxia and/or asphyxia, hypoxic-ischemic encephalopathy, congenital heart disease, inborn errors of metabolism, sepsis, nephrotoxin exposure, patent ductus arteriosus, and extracorporeal therapies.

Here, we present a case report of a term infant presenting with a decrease in urine output and signs of dehydration. Sonography is used as a primary modality to evaluate the kidneys for any structural deformities. A normal sonographic image of the kidneys of a neonate would show hypoechoic pyramids of the medulla. Nevertheless, the entity of less frequently occurring neonatal transient renal failure with renal medullary hyperechogenicity has been linked to severe perinatal renal damage, kidney abnormalities, or nephrocalcinosis [4].

## Case presentation

A five-day-old full-term male infant weighing 2.4 kg presented with signs of decreased skin turgor and urine output. The mother gave no history of any drug intake either when she was pregnant or while nursing the newborn. The passage of urine recorded in the bag was at the rate of 1.0 mL/kg/hour. There were no other significant findings on the physical examination.

After obtaining informed consent from the patient’s guardian, laboratory and radiological investigations were performed. Laboratory data on admission showed hemoglobin of 16 g%, blood urea of 325 mmol/L (slightly raised), serum creatinine of 6.9 µmol/L, serum sodium of 166 mmol/L, and serum potassium of 4.0 mmol/L. A routine urine examination showed three to four pus cells in the sample (normally <1 pus cell should be present).

Ultrasound findings

**Figure 1:**
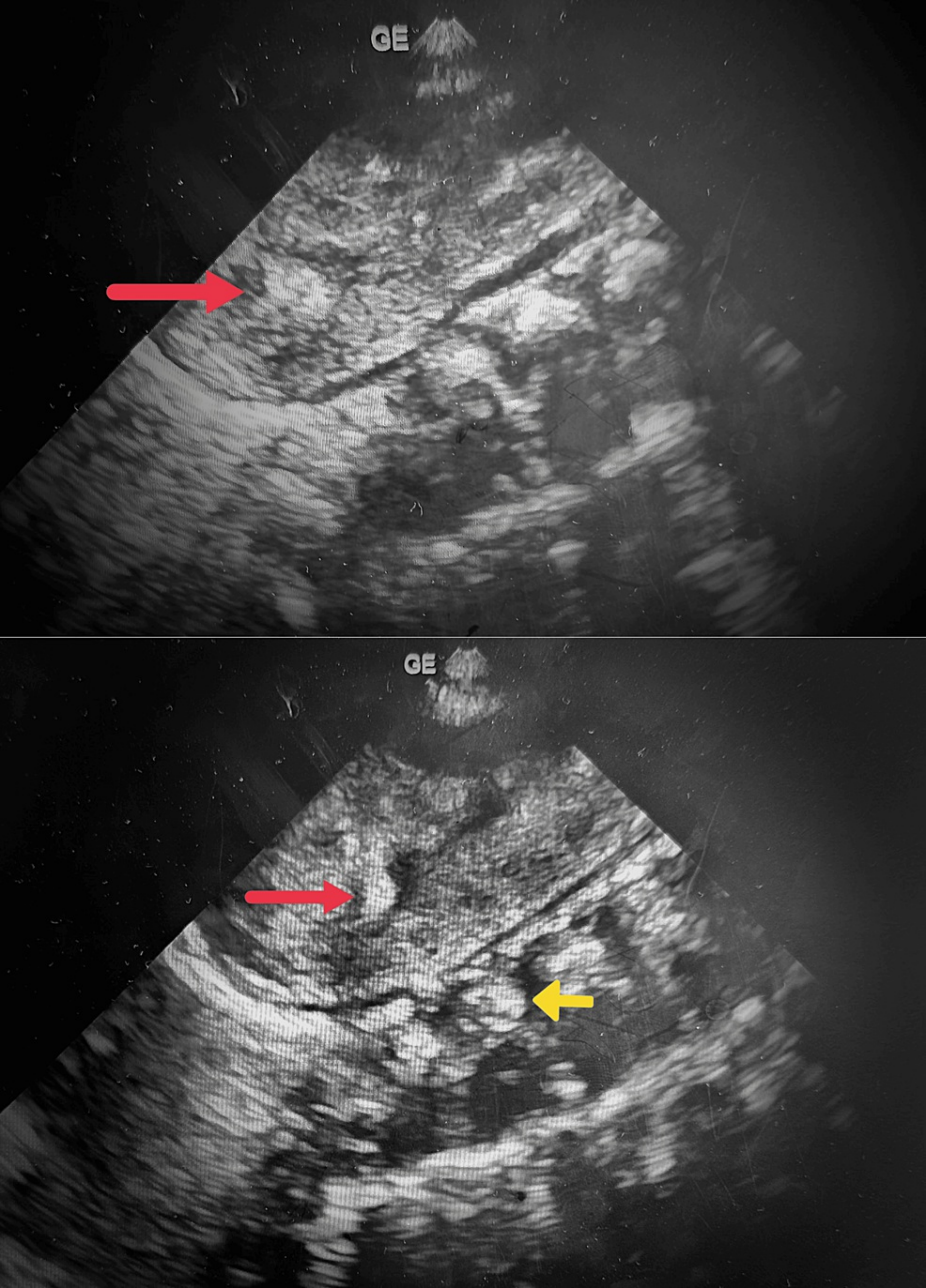
Grayscale B-mode image of the left and right kidney showing hyperechoic medullary pyramids (red arrows) and hyperechoic vertebral bodies giving posterior shadowing (yellow arrow).

In the right and left kidneys, ultrasound revealed a normal kidney size of 4.8-5 cm in length, normal cortical echogenicity, hyperechogenic pyramid tips with a diminishing gradient toward the base of the pyramid, and perhaps dilated renal pelvis. There was no acoustic shadowing (Figure 1).

**Figure 2:**
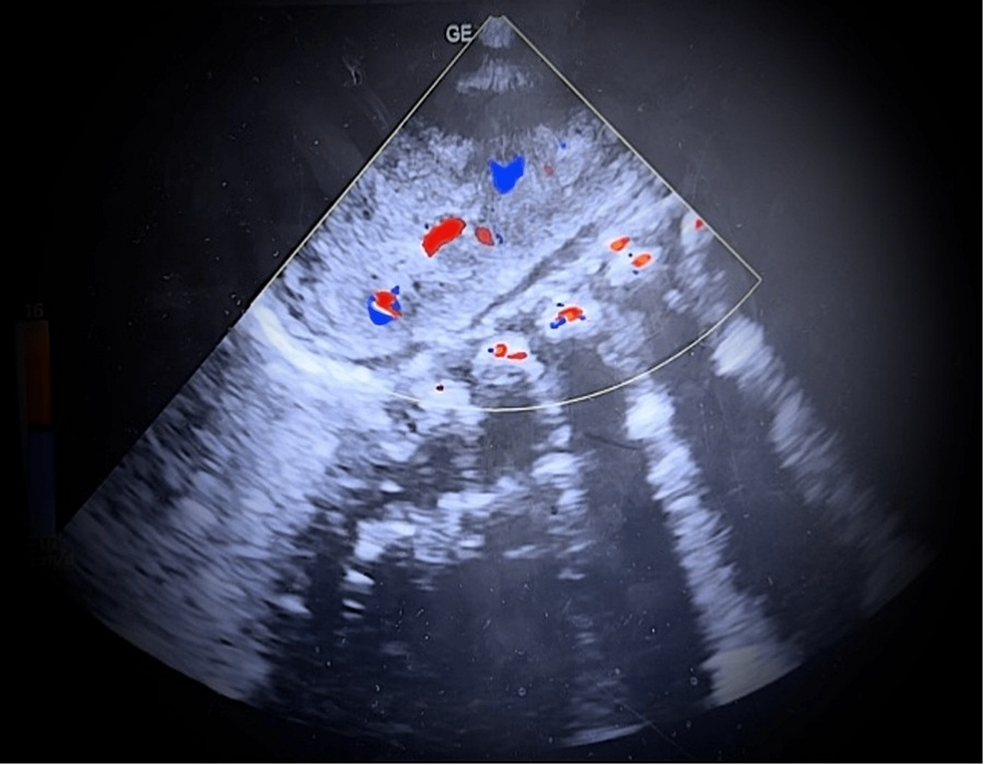
Color Doppler image of the left kidney showing the “red, white, blue” sign (artery, pyramid, vein).

On the Doppler ultrasound, there was also a distinguished “red, blue, white” sign representing renal vasculature with intervening medulla of the kidney (Figure 2).

Follow-up

**Figure 3:**
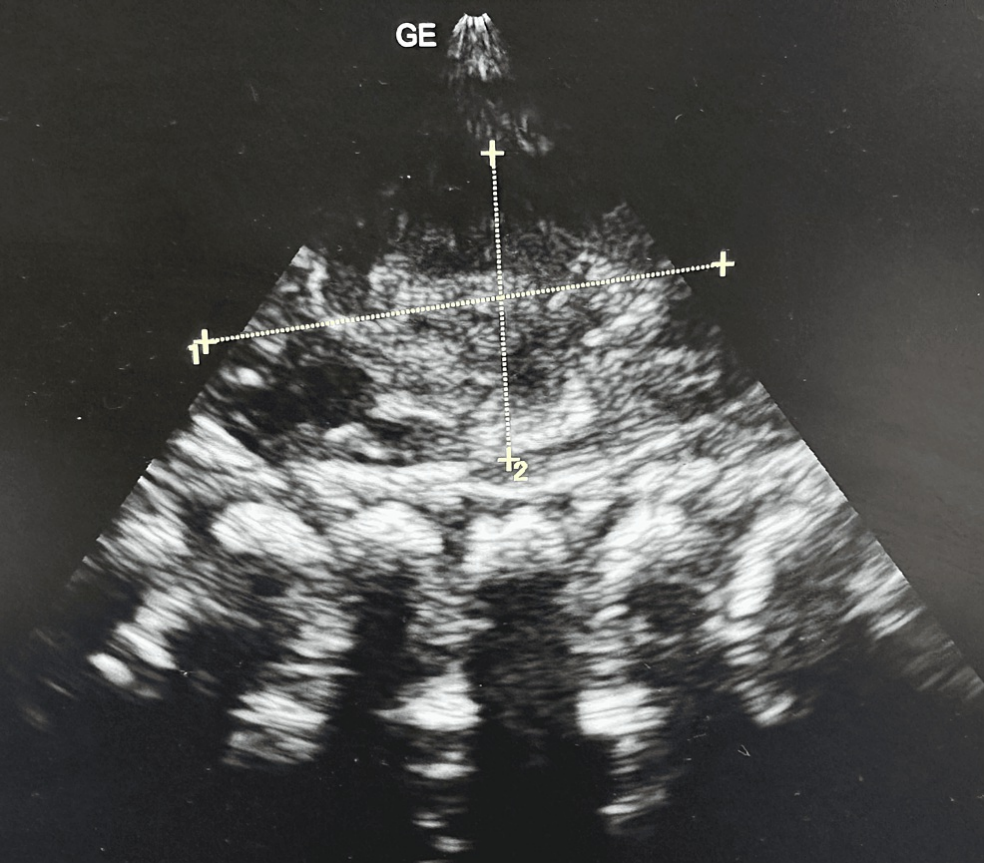
Follow-up grayscale ultrasound image showing normal anechoic renal medulla.

The neonate was started on intravenous rehydration. Subsequently, the oliguria resolved, with a urine output of 3.0 mL/kg/hour on day seven. At the time of discharge, serum creatinine was 0.6 µmol/L. After two weeks, a second renal ultrasound revealed no pelvic dilatation and normal parenchymal echogenicity (Figure 3).

## Discussion

The usual sonographic image of a neonatal kidney shows a hypoechogenic medulla compared to the cortex. A large study involving 28 neonates conducted by Makhoul et al. [4] concluded that in 0.1% of term live-born newborns, the separate entity of transitory renal dysfunction due to precipitation of some endogenous substances, namely, uric acid and Tamm-Horsfall protein, with renal medullary hyperechogenicity occurred, which was in contrast to most of the cases where this hyperechogenicity was due to other causes such as nephrocalcinosis and in those undergoing furosemide therapy.

Neonatal nephrocalcinosis has been observed in William’s syndrome, distal renal tubular acidosis, neonatal Bartter syndrome, oxalosis, vitamin D therapy, and primary hyperparathyroidism [5]. Several criteria can be used to distinguish between these disorders and transient benign renal medullary hyperechogenicity [6], which include physical examination, lab investigations, and ultrasonography findings in neonates who do not show any symptoms other than dehydration, with raised creatinine and uric acid levels. Sonography reveals hyperechoic tips of the medullary pyramids without acoustic shadowing [7].

Treatment includes intravenous rehydration with fluids and complete watch with follow-up. Subsequent ultrasonography taken after 10 to 14 days shows echolucent renal pyramids and normalization of laboratory values.

## Conclusions

As stated by various other studies as well, the above sonographic finding of hyperechoic medullary renal pyramids found in neonates presenting with dehydration is not indicative of intrinsic renal disease rather it subsides without intervention, if physiologic, or with rehydration, if due to hypernatremic dehydration. Thus, it is imperative for the treating physician and diagnostician to know about this physiological variant to manage the neonate conservatively. On follow-up, the patient’s parents were satisfied with the treatment outcomes.

## References

[1] Coleman C, Tambay Perez A, Selewski DT, Steflik HJ (2022). Neonatal acute kidney injury. Front Pediatr.

[2] Zappitelli M, Ambalavanan N, Askenazi DJ (2017). Developing a neonatal acute kidney injury research definition: a report from the NIDDK neonatal AKI workshop. Pediatr Res.

[3] Starr MC, Charlton JR, Guillet R (2021). Advances in neonatal acute kidney injury. Pediatrics.

[4] Makhoul IR, Soudack M, Smolkin T (2005). Neonatal transient renal failure with renal medullary hyperechogenicity: clinical and laboratory features. Pediatr Nephrol.

[5] Schell-Feith EA, Kist-van Holthe JE, van der Heijden AJ (2010). Nephrocalcinosis in preterm neonates. Pediatr Nephrol.

[6] Bouwman A, Verbeke J, Brand M, Bökenkamp A (2010). Renal medullary hyperechogenicity in a neonate with oliguria. NDT Plus.

[7] Nakamura M, Yokota K, Chen C, Taniguchi N, Izumi A, Kawai F, Itoh K (1999). Hyperechoic renal papillae as a physiological finding in neonates. Clin Radiol.

